# T Cell Subsets During Early Life and Their Implication in the Treatment of Childhood Acute Lymphoblastic Leukemia

**DOI:** 10.3389/fimmu.2021.582539

**Published:** 2021-03-04

**Authors:** Shanie Saghafian-Hedengren, Eva Sverremark-Ekström, Anna Nilsson

**Affiliations:** ^1^Division of Paediatric Oncology and Paediatric Surgery, Department of Women's and Children's Health, Karolinska Institutet, Stockholm, Sweden; ^2^Department of Molecular Biosciences, The Wenner-Gren Institute, Stockholm University, Stockholm, Sweden

**Keywords:** T cells, childhood, leukemia, chemotherapy, immunotherapy

## Abstract

The immune system plays a major role in recognizing and eliminating malignant cells, and this has been exploited in the development of immunotherapies aimed at either activating or reactivating the anti-tumor activity of a patient's immune system. A wide range of therapeutic approaches involving T lymphocytes, such as programmed cell death protein ligand-1 (PDL-1) inhibitors, cytotoxic T-lymphocyte-associated protein-4 (CTLA-4) blockers, and CD19-targeted T-cell therapy through chimeric antigen receptor (CAR)-T cells or CD19/CD3 bi-specific T-cell engagers, have been introduced to the field of oncology, leading to significant improvements in overall survival of adult cancer patients. During the past few years, the availability and approval of T-cell based immunotherapies have become a reality also for the treatment of childhood cancers. However, the distribution, ratio of regulatory to effector cells and the quality of T-cell responses early in life are distinct from those during adolescence and adulthood, raising the possibility that these differences impact the efficacy of immunotherapy. Herein we provide a brief overview of the properties of conventional T cell subsets during early life. Focusing on the most common cancer type during childhood, acute lymphoblastic leukemia (ALL), we describe how current conventional therapies used against ALL influence the T-cell compartment of small children. We describe early life T-cell responses in relation to immunotherapies engaging T-cell anticancer reactivity and present our opinion that it is not only immaturity of the adaptive immune system, but also the impact of an immunosuppressive environment that may prove disadvantageous in the setting of immunotherapies targeting pediatric cancer cells.

## Introduction to Cancer Immunology and Immunotherapies

Initially in the field of cancer immunology, Burnet proposed the immunosurveillance theory, suggesting that lymphocytes continuously scan and eliminate transformed cells to maintain the cells of the host in homeostasis (1). Evidence in favor of this theory came from observations in mice, where immunodeficient strains displayed high rates of spontaneous tumor development as well as higher susceptibility to induced tumors. An increased incidence of tumors has also been noted in immunosuppressed patients (2). This theory was however challenged, since many patients develop cancer in the absence of overt immunosuppression or immunodeficiency. As tumor cells are able to escape immunosurveillance, the theory was refined and the concept of “cancer immunoediting” emerged. This describes three key immunological features to combat immunogenic malignant cells: the tumor elimination phase by natural killer and T cells (incorporates immunosurveillance); the equilibrium phase between immune and malignant cells; and the escape phase resulting in clinically overt cancer as the host's immune system fails to eradicate cancer cells (3). Furthermore, in 2011 Hanahan and Weinberg (4) proposed that one additional hallmark of cancer was the ability to avoid immune destruction through the secretion of immunosuppressive factors from cancer cells and/or the recruitment of inflammatory or regulatory T cells (Tregs) to the tumor site.

During the last decades a wide range of immunotherapeutic approaches have been introduced for cancer treatment, such as programmed cell death protein ligand-1 (PDL-1) inhibitors, cytotoxic T-lymphocyte-associated protein-4 (CTLA-4) blockers, CD22-targeting therapy with Inotuzumab ozogamicin and targeted T-cell therapy through chimeric antigen receptor (CAR)-T cells or bi-specific T-cell engagers leading to significant improvements in overall survival for a number of cancer patients. Similarly, in pediatric oncology, the last decade has seen some promising results from immunotherapy in children with relapsing disease. In this brief review, we will focus on acute lymphoblastic leukemia (ALL), the most common childhood cancer. We will describe the attributes of conventional T cells that are present during the early life of humans, and place these in the context of CD19-targeted T-cell immunotherapies against ALL.

## The Human T Cell Compartment at Different Ages

The human new born immune system faces several challenges immediately after birth. During the very first years of life, the immune system encounters a myriad of different antigens and has to decide whether to react (against pathogens or transformed cells) or develop tolerance (against innocuous antigens or self-antigens). This balancing act has to continue throughout our entire lifespan in an environment with constantly changing antigens. In contrast to mice, human T cell development starts early during gestation (5). During the second trimester a developmental shift in lymphopoiesis occurs and the T-cell receptor (TCR) repertoire becomes diversified (5). New born children have a complete set of single CD4^+^ (helper) and CD8^+^ (cytotoxic) T cell subsets at birth, even when born pre-maturely (5–7). As a result of a marked expansion of recent thymic emigrants and naïve cells, absolute T cell numbers increase rapidly after birth to peak during the first month of life, followed by a gradual decrease during the first years of life to reach adult levels (5, 6). During this formative stage, T cells play a key role in combatting pathogens and establishing a memory T cell pool. Both the absolute numbers and frequency of Tregs are abundant in infants, where they are critical for developing tolerance. These cells decline from 5 to 8 years of age, reaching similar numbers to those of adults over time (7, 8). Focusing on findings from studies in humans, in the following section we will touch upon key attributes that distinguish conventional T cell populations in young children from those in adults.

Immune system competence is connected to the diversity of the host's T cell pool (9), which subsequently increases an individual's probability to recognize invading pathogens. This T cell diversity relies on the presence of a naïve T cell pool which is formed through thymopoiesis. Overall, it appears that naïve T cells in young children are not necessarily less responsive, but rather programmed to respond differently to activation signals compared to adults. Recent evidence suggests that neonatal T cells are biased toward non-specific defense mechanisms, which are less dependent on TCR recognition, while having an elevated capacity to react to inflammatory and danger signals, which are in part facilitated through the expression of innate receptors [reviewed in (10)]. Thymic output can be assessed on the basis of cell markers for recent thymic emigrants, such as surface CD31, or the presence of signal joint T-cell receptor excision circles (sjTRECs), both of which are higher in children (11, 12). By means of proliferation ability, naïve T cells in humans can be maintained through homeostatic peripheral expansion by IL-7 and IL-15 cytokines, which act pre-ferentially on CD4^+^ and CD8^+^, respectively (13, 14). IL-7-induced cell division does not, however, lead to helper T cell differentiation, as is the case with CD8^+^ T cells following IL-15 exposure and activation (15, 16). A high constitutive telomerase activity has been shown to protect neonatal T cells from proliferative stress and exhaustion (13), a phenomenon more commonly noted in adult T cells. Another interesting observation is that the increased apoptotic potential in human neonatal T cells (14, 15) can be reverted by cytokines acting via the IL-2 receptor γ-chain (17–19).

The effector T cell functions in early life are characterized by decreased IFN-γ but higher type 2 cytokine and CXCL8 production (20, 21), which in part have been explained by the epigenetic conformation of IL-13 (22) and IFN-γ loci (23) in neonatal CD4^+^ T cells. The transcriptome and epigenetic profiles, along with functional readouts from cord blood and adult naïve CD8^+^ T cells, corroborated that neonatal CD8^+^ T cells are more innate-like by displaying, among others, less cytotoxic functions, while being more prone toward producing antimicrobial peptides and reactive oxygen species (ROS) (24). It is not clear if costimulatory capacity through CD40 ligand on T cells is defective (25–27), or if a differential activity of the transcription factor NFAT (28), which affects T-cell costimulatory capacity and IFN-γ production, is present during early childhood years.

The involution of the human thymus begins already during the first year of life (29–31) and proceeds at a steady rate of 3% thymic volume loss until ~50-years of age, after which the involution rate decreases somewhat to a new stable level in the elderly (32, 33). If the thymus already starts to involute after birth (29) then the question is how a naïve T cell pool, that can react to new antigen challenges, is maintained from birth onwards in humans? Farber and colleagues have made significant contributions to advancing our understanding in this field. In one of their recent studies, that characterized naïve T cells in various secondary lymphoid tissues in humans (34), it was demonstrated that a dramatic decline in thymic output occurs rather from middle age onwards, whereby a four-fold decrease of double positive thymocytes is noted. Furthermore, it was shown that each lymphoid organ contains a unique set of naïve T cell clones which can expand, especially in those past 40 years of age, suggesting that naïve T cells can be sustained through *in situ* homeostasis and retention in lymphoid tissues.

The distribution and activity of T cells across tissues appear to be influenced by age. Upon comparing immunophenotypes of CD4^+^ and CD8^+^ T cells in tissues from pediatric and young adult organ donors (12), a higher frequency of naïve T cells was found across all pediatric tissues, including the lung and intestine, compared to adult tissue. Memory T cells in children, on the other hand, were found at local mucosal sites, such as lungs and intestine, but less frequent in blood and secondary lymphoid organs, while in adults both lymphoid and mucosal tissues contained high proportions of CD4^+^ and CD8^+^ memory T cells. In general, lymphoid pediatric T cells produce fewer effector cytokines (IL-1, IL-4, and IFN-γ) than adult tissue T cells, with the exception of pediatric memory T-cells derived from the intestinal mucosa (which secrete proinflammatory cytokines at similar levels to those in adults). Tissue resident memory T cells are key to providing local protective immunity. When examining tissue memory T cells for markers associated with tissue retention, pediatric mucosal samples contain a lower frequency of T cells expressing both the activation marker CD69 and the integrin CD103 compared to adults, which suggests that memory T cells in early life have not yet fully acquired a tissue resident phenotype (12). In response to respiratory tract infection, memory T-cell formation appears more limited during infancy, as evidenced by the accumulation of more terminally differentiated (TEMRA) CD8^+^ T cells in the lungs of younger patients, whereas the less-differentiated tissue-resident memory CD8^+^ T cells were more often seen in older children (35). Corresponding data in pediatric populations on the characteristics of tissue memory helper T cells following infection are lacking.

The microbiome is an important factor for T-cell compartment maturation, including Tregs (36, 37). There are various ways for commensals to shape the immune system, both directly with the adjacent immune system in the gut as well as on distance via metabolites (38), microbial compounds (39), and potentially also extracellular microvesicles (40). Tregs are a subset of CD4^+^ T cells identified through the master transcription factor, forkhead box protein P3 (FoxP3), with essential roles in maintaining self-tolerance and dampening immune responses (41). Peripheral Tregs represent around 5–10% of CD4^+^ T cells in cord blood (42, 43). They comprise 30–40% of the CD4^+^ T cells in pediatric tissues and 1–10% in adult tissues (12). Tregs display an entirely different TCR repertoire to other T cell populations and also exhibit a higher cell turnover compared to naïve T cells (44). Functionally, the depletion of Tregs in pediatric lymphoid and mucosal tissue results in potentiated T cell proliferation and cytokine release (12). By contrast, depletion of Treg cells in adult tissue does not alter T cell activation. Based on differential CD45RA expression, two distinct Treg subsets termed activated and resting have been identified among the neonatal FoxP3^+^ CD4^+^ T cells. These subsets are functionally distinct, such that activated Tregs are terminally differentiated and rapidly die after exerting suppression while resting Treg cells proliferate, become CTLA-4^+^ FoxP3^hi^ and replenish the activated Treg pool (45). Both activated and resting Tregs increase over the first 6 months post-natally, but subsequently it is mainly the proportion of activated Tregs that increase (46).

To summarize, the significant observations of increased Treg/effector memory T cell ratios across various tissues in infants compared to adults, together with the elevated T cell response following Treg depletion in children, indicate that-rather than being intrinsically defective-infant T cells may simply be more inhibited than their adult counterparts in allowing for immune maturation and tolerance. We speculate that this may have important implications for T-cell based immunotherapies designated for use in younger children.

## Childhood all and Current Treatment Regimens

The global incidence of childhood cancer is estimated at ~152.8 per million person-years in 0 to 19-year-olds, with a slightly higher incidence in the age groups 0 to 4-year-old and 15 to 19-year-old (47). Childhood cancers span diverse clinical and biological tumor entities, including leukemia and lymphoma, bone and soft tissue sarcoma, central nervous system tumors, retinoblastoma, neuroblastoma, liver and kidney tumors, germ cell tumors, and additional rare cancers. Childhood leukemia represents ~30% of all childhood cancer cases, with ALL accounting for 80% and acute myeloid leukemia (AML) for 15–20% of cases (48). There are only rare cases of chronic myeloid leukemia (CML) in children. Dramatic improvements in the overall ALL survival rates have been accomplished over the last decades, with outcomes approaching 90% (49). However, progress has been slower for certain patient subgroups, such as infant-ALL, Down's syndrome and children with AML. For the purpose of this review, we will from now on focus our discussion on ALL.

Childhood ALL includes a number of subtypes defined by cell lineage (B- or T-cell), differentiation status and genetic alterations (50). These biological characteristics differ by age distribution and clinical outcome and are, therefore, used for tailoring therapy to the predicted risk of relapse. The majority of children with ALL are classified as non-high risk (HR) patients and stratified to standard (S) or intermediate (I) risk protocols at diagnosis. HR characteristics at diagnosis include age >10 years, disease onset with a WBC >50,000/μl, and the presence of known poor genetic alterations (51). Treatment of childhood ALL consists of various chemotherapy components (induction, consolidation, intensification, and maintenance with CNS prophylaxis), with treatment intensity increasing slightly between children in the standard (SR) and those in the intermediate (IR) risk groups, applied according to the respective treatment protocols. In children presenting with HR-features, treatment entails a more aggressive multimodal chemotherapy and may include allogeneic stem cell transplantation (HSCT) to achieve cure. The overall treatment period varies between 2 and 2.5 years for the majority of international protocols. Despite the risk-adapted tailoring of therapy, the most important prognostic factor is how quickly and to what extent the tumor burden decreases once therapy is initiated. Response to treatment is measured by flow cytometry (and/or PCR) after ~4 weeks of induction therapy and, at the same time, the presence of minimal residual disease (MRD, i.e., leukemic cells) in the bone marrow compartment is evaluated. Absence or persistence of MRD at defined treatment points will stratify the patient to the final risk group and treatment intensity. However, in 10–15% of those children who achieve remission, the disease will return and relapse. The precise mechanisms behind treatment failure are not fully elucidated. To date, it is believed that ALL relapses emerge from subclonal populations that share some of the mutations identified in the dominant population at diagnosis, but also some additional new mutations associated with chemoresistance (52–54). Whether childhood ALL relapses are in any way related to the emergence of immune regulatory cells suppressing anti-cancer immunity (55) remains to be clarified.

## T Cell Immunity at Cancer Diagnosis and Post-Therapy in Children With All

Overall, little is known about the normal T-lymphocyte population in the pediatric ALL setting. Several studies have reported increased T- cell proportions (both single CD4^+^ and CD8^+^) including Tregs at the diagnosis of childhood B-cell ALL (56, 57). Studies have shown that children at the time of their ALL diagnosis have both higher proportions and absolute cell numbers of bulk CD4^+^ and CD8^+^ T cells in peripheral blood as well as increased proportions of Tregs compared to age-matched controls (58, 59). The proportion of Tregs remained higher throughout the first 4–5 weeks of induction therapy. In addition, a study investigated the phenotype of (non-malignant) T cells present in the bone marrow at the time of diagnosis in 39 children with ALL. Data showed that a higher CD4:CD8 ratio correlated with a better treatment response at day 15, and that this association was caused by non-Tregs (60). However, the significance of Tregs in the anti-cancer response or the pathogenesis of childhood ALL is far from clear.

The use of more intense chemotherapy protocols has led to a dramatic improvement in prognosis, unfortunately accompanied by both acute- and long-term toxicity that cause significant treatment-related morbidity. Several long-term defects in humoral immunity in children treated for ALL have been noted [reviewed in (61)], but how chemotherapy affects conventional T cell subsets and their functions is less well-studied (summarized in Table 1). The general understanding is that T cells remain more resistant toward chemotherapy than B cells (61, 67). Studies have shown that the T cell recovery at bulk level is delayed post-chemotherapy and that this delay is less pronounced in toddlers and young children (62, 70). It has also been suggested that helper T-cells have a longer recovery time than cytotoxic T-cells (65, 66, 71), an effect that has shown to be more prominent in more intense chemotherapy settings (63, 68). There is also evidence of a lower proportion of T cells in peripheral blood during chemotherapy, with a pronounced effect on naïve compared to memory T-cells, the latter remaining overrepresented during a 3-year period of chemotherapy (64, 69). One may speculate that thymic output of naïve T cells is severely affected by conventional chemotherapy including steroids, but data in favor of this is as yet incomplete in the literature as opposed to that for post-haematopoietic stem cell transplantation (HSCT) [reviewed in (72)].

**Table 1:** Peripheral T cell populations in children during and after ALL therapy.

**References**	**Patients** **(*n*)**	**Diagnosis, age** **(years)**	**Sampling time**	**T cell subsets**	**Results**
Alanko et al. (62)	14	ALL Age 3–18	After cessation of therapy; 0, 1, 3, 6, 9, and 12 months	CD4^+^	Subnormal at *t* = 0 but normalized at 6 months in children 7–18 years old In children 3–6 years CD4^+^T cells normalized within 1 month
				CD8^+^	Subnormal at *t* = 0 but normalized at 3 months in children 7–18 years old. In children 3–6 years CD8^+^ were within normal range at cessation of therapy
Ek et al. (63)	31 40	ALL (SR, IR, HR) Age 3–19 HC Age 2–16	After cessation of therapy; at 1 or 6 months	Bulk CD4^+^	59% (median) at 1 month and 65% (median) at 6 months post-therapy in HC 54% (median) at 1 month and 57% (median) at 6 months post-therapy in HC. Naïve CD4^+^ cells were 42 and 60% (median values) at 1 and 6 months
				CD8^+^	73% (median) at 1 month and 77% (median) at 6 months post-therapy in HC. No differences in naïve or memory CD8^+^ T cells compared to controls
Haining et al. (64)	73	ALL Age 1–17	At diagnosis and during the 24 months of therapy	CD4^+^ and CD8^+^	55% of patients showed <10th percentile of CD4^+^ T cells for age and 77% showed <10th percentile of CD8+ T cells. No significant recovery occurred during the 24 months of therapy
				Naïve CD4^+^ and CD8^+^	55% median of reference value for age 44% median of reference value for age TRECs were significantly lower in ALL compared to healthy controls at diagnosis and during therapy
				Memory CD4^+^ and CD8^+^	CD4^+^ memory cells remained elevated during the study period compared to the reference value for age. CD8^+^ memory cells were elevated at diagnosis but normalized compared to the reference value for age
Mazur et al. (65)	38 30	ALL Age 5–18 HC Age 5–18	After cessation of therapy; 1, 4, 7, 10, and 13 months	CD4^+^	50% median of reference value for age at 1 month and 76% of age reference at 12 months
				CD8^+^	86% median of reference value for age at 1 month and 100% of age reference at 13 months
				Naïve CD4^+^ and CD8^+^	At 13 months, 55 and 80% of reference value for age, respectively
				Memory CD4^+^ and CD8^+^	At 13 months, 61 and 100% of reference value for age, respectively
van Tilburg et al. (66)	31	ALL (*n* = 21) (non-HR and HR), AML (*n* = 4), lymphoma (*n* = 6) Age 4–16	1–8 samplings over a variable period after end of chemotherapy	Naïve CD4^+^	36% (median) of reference value for age at end of chemotherapy Normalized 3–6 months. Total CD4^+^ TREC increased concomitant with CD4^+^ cells
				Memory CD4^+^	35% (median) of reference value for age at end of chemotherapy. Increased in the first 3 months but remained reduced over the study period
				Naïve CD8^+^	57% (median) of reference value for age at end of chemotherapy Normalized after 6 months
				Memory CD8^+^	41% (median) of reference value for age at end of chemotherapy. Remained low during reconstitution.
				Effector CD8^+^	11% (median) of reference value for age at end of chemotherapy. Regained normal values >2 years after cessation
van Tilburg et al. (67)	140	ALL (SR, MR, HC) Age 1.5–18	Samples taken on days 11, 20, 39, 57, and at end of treatment and 1-year post-therapy	CD4^+^	27% (median, SR patients) and 31% (median, MR patients) of reference value for age at end of chemotherapy; 98% (median, SR patients) and 98% (median, MR patients) 52 weeks after end of chemotherapy
				CD8^+^	34% (median, SR patients) and 41% (median, MR patients) of reference value for age at end of chemotherapy; 113% (median, SR patients) and 97% (median, MR patients) 52 weeks after end of chemotherapy
Koskenvuo et al. (68)	28	ALL (SR and IR) Age 4–19	Samples taken at 0, 3, 6, 9, 12, 18, and 24 months after cessation	CD4^+^, CD8^+^ and Treg	Lower CD4^+^ and CD8^+^ T cells in IR children at cessation of therapy. After that, no significant differences in any sub-population was noted between SR and IR children
Das et al. (69)		ALL (SR+HR) Age 1–10	Samples taken at 0 and after each protocol cycle	Naïve CD3+ CD4+/CD8+ ratio	Lower percentage of naïve T cells at diagnosis, which declined further over time Lower ratio at *t* = 0 and declining compared to normal donors

*ALL, acute lymphoblastic leukemia; HC, healthy controls; SR, MR, IR, HR, standard, medium, intermediate, high risk; AML, acute myeloid leukemia; Treg, regulatory T cell*.

HSCT has been a curative option for a number of years for pediatric patients with relapsed ALL. Children considered for CD19-targeted immunotherapy are to a large extent in post-HSCT and possibly in their immune-reconstitution phase. There are several parameters that will impact on the kinetics of T-cell recovery after HSCT, such as the conditioning regimens, donor type and age, graft manipulation, type of graft-vs-host disease (GvHD), as well as treatment and prophylaxis [reviewed in (73)]. In children followed for T cell recovery after HSCT for hematological malignancies, studies suggest that recent thymic emigrants and TRECs recover within 6–12 months post-HSCT (11, 74, 75). Also, interventions aimed at treating GvHD itself appear to impact on thymic recovery and the naïve T cell pool, where an association between systemic steroid treatment and delayed CD4^+^ T-cell recovery has been noted (74). However, the current picture of post-HSCT dynamics is that Tregs are normalized within weeks followed by the recovery of memory CD4^+^ and CD8^+^ T cell within months. In contrast, the recovery of naïve T cell pool can take years, likely as this population relies on thymic output and not on peripheral homeostasis and expansion (76). Finally, while chemotherapy and HSCT have been shown to clearly impact the proportions of circulating T cells, there are almost no studies that systematically assess the functional responses of T cells (in particular the memory population) following ALL treatment. Although the proliferative potential of T cells to varicella zoster virus has been concluded as intact (64), these findings have not been corroborated by other cellular functions, such as cytotoxicity.

## Introduction of CD19-Targeted Immunotherapies for Childhood All

The introduction of new therapies for pediatric patients has been slow compared to adults, but in recent years an acceleration in both the availability and approval of novel agents for the treatment of childhood B-ALL has occurred. At least two of the most promising approaches–the CD19/CD3 bi-specific T-cell engager, blinatumomab, and the CD19-chimeric antigen receptor T (CAR-T) cell therapy–engage the patient's own T cells to bind and lyse the CD19^+^ B-ALL cells. During therapy, T cells are activated to unleash an aggressive immune response toward the leukemia, but also an inflammatory response that may result in severe cytokine-release syndrome and neurological toxicity. Both blinatumomab and CAR-T cell therapy have shown promising results in the treatment of relapsed/refractory pediatric B-ALL (77). von Stackelberg et al. (78) were among the first to show the efficacy of blinatumomab in heavily pre-treated pediatric ALL patients. Last year, a randomized phase 3 trial of blinatumomab vs. standard post-induction chemotherapy in high risk (early) first relapse of pediatric B-ALL showed a clear treatment benefit in the group of children who received blinatumomab: less toxicity, higher remission rates at day 29, and more children becoming eligible for HSCT (79). This study will probably shift the clinical therapeutic decisions in high risk ALL relapses in favor of immunotherapy over standard chemotherapy.

The first reported patient treated with second-generation anti-CD19 CAR T cells was an adult with refractory CLL who, after one infusion of CAR T cells, achieved remission that was sustained for 10 months (80). This was soon followed by several pediatric phase 1 trials with different CAR T cell products [reviewed in (77)]. In 2017, an important study was published in which the CAR T cell product was refined by introducing a consistent 1:1 CD4 to CD8 ratio, uniform CAR expression and limited T cell differentiation in the culture system (81). In total, 43 children and young adults with relapsed ALL were then infused and the intent-to-treat analysis showed a promising 89% response rate. In the pivotal global registration trial for tisagenlecleucel (82) the final analysis showed an outcome for 75 infused pediatric and young adult patients (total study cohort 107 screened and 92 enrolled patients). Intent-to-treat analysis showed a 66% response rate in this heavily pre-treated group of patients. In August 2017, the FDA approved tisagenlecleucel as the first commercial product for relapsed/refractory B cell malignancies in patients up to 25 years of age (83). However, even though both CAR-T cell therapy and bi-specific T-cell engagers show promising results, a significant proportion of patients do not respond or suffer severe toxicity. Even in responders, ALL immunotherapy, unless based on long-term persistent CAR T cell products, is mostly seen as a bridge to HSCT and potential cure. Whether CAR T cell therapy can replace HSCT in subsets of pediatric ALL patients is one of the most relevant questions in ALL HR therapy and will need to be addressed in prospective trials.

## Future Patient Selection for Immunotherapy in All

While immunotherapy is also a realistic option for children, one question that remains to be answered is how to improve the selection of patients for immunotherapy. Currently, T-cell expansion once immunotherapy is initiated can, to some extent, predict treatment response (84). Also, persistent B cell aplasia is a marker for long term response after anti-CD19 CAR-T cell and CD19/CD3 bi-specific T-cell engager therapy as long as CD19 expression is preserved on leukemic cells. But can we improve the selection of patients for immunotherapy upfront? In one recent study lymphocyte subsets in peripheral blood of adult patients receiving blinatumomab was investigated and responders were compared to non-responders (85). The most significant marker discriminating responders from non-responders was the percentage of Tregs, while there was no correlation in the absolute number of lymphocytes, CD4^+^ or CD8^+^ T cells, for either the naïve or effector populations. As Tregs are more abundant during childhood, similar studies in pediatric ALL patients are warranted. Recent data suggest that the T cell responses to blinatumomab may also be modulated by the expression of inhibitory molecules on the B-ALL leukemic cells (86). First, a number of co-stimulatory and inhibitory molecules regulating T cell responses were assessed by flow cytometry on patient-derived bone marrow blasts. PDL-1 was the most prominent inhibitory marker on these primary blasts while CD80 was the most highly expressed stimulatory marker. Interestingly, PDL-1 expression was significantly higher on patient leukemic cells in blinatumomab non-responders compared to responders and healthy controls. In the same report, a case regarding a 12-year-old girl with refractory ALL was presented. The patient had previous non-response to monotherapy with blinatumomab, but when combined with a PD-1 blocking antibody a partial anti-leukemic T cell effect was noted. This is in keeping with findings in adults, where blinatumomab was shown to activate Tregs to suppress effector responses by cytotoxic T cells, with the conclusion that therapeutic removal of Tregs could provide a means for converting blinatumomab non-responders to responders (85). Altogether, these studies show the potential of a detailed phenotypic characterization of T cell populations in patients that proceed to immunotherapy, which could possibly predict response and enable further tailoring of immunotherapy.

## Conclusions and Future Directions

**Figure 1:**
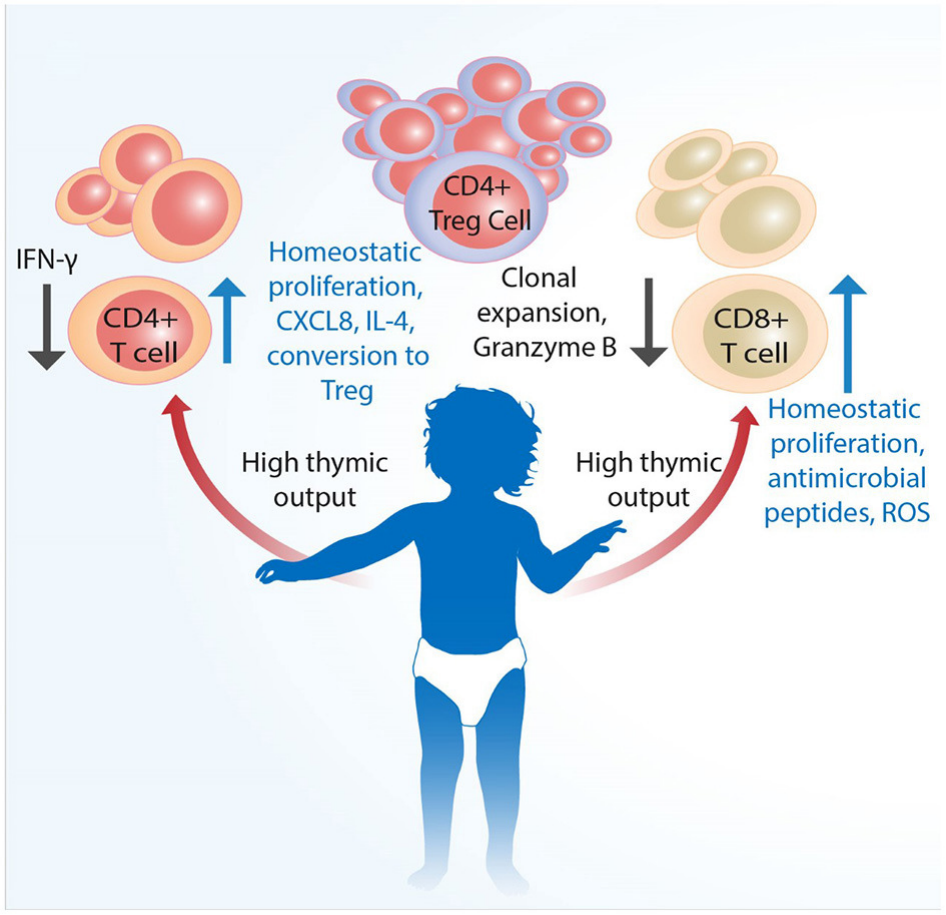
Infant T cells are qualitatively distinct from adult cells. Conventional T cells develop in the thymus, the size as well as output of which is largest at birth. Mature single CD4^+^ and CD8^+^ cells are proportionally less than Tregs across various tissues. Although infants have substantial populations of fully developed single CD4^+^ and CD8^+^ cells T cells from birth, these are predominantly naïve and more prone to type 2 cytokine and innate-like responses following activation. Naïve CD4^+^ T cells have higher propensity to develop into FoxP3^+^ Tregs to actively promote self-tolerance. An increased susceptibility to infection in neonates is likely not a result of only immaturity of adaptive immunity, but also that of qualitatively different functional capacity and higher level of immunosuppression, which together may negatively impact the eradication of malignant cells.

The generation, function, and regulation of immune responses by T cells during early life in humans are only partially understood. The fetal environment requires that the immune system tolerates maternal alloantigens. Following birth, a sudden shift in exposure to environmental antigens, including gut commensal bacteria, necessitates adaptation of immune responses to suit early life. Even though neonates and infants have substantial populations of T cells from birth (87), they are predominantly naïve, expressing distinct patterns of homing receptors compared to adults, and as a rule more prone to producing regulatory (14, 88) and innate-like (24) responses following activation (Figure 1). As a consequence of maintaining self-tolerance (including toward gut commensals) and to avoid immunopathology, the new born will be relatively susceptible to infections. This would be in keeping with the “disease tolerance” posit (89), which suggests that an increased susceptibility to infection in neonates is not a result of immaturity but rather one of an immunosuppressive environment, which is in part an active defense strategy likely to be disadvantageous for the young host with malignant cells.

**Figure 2:**
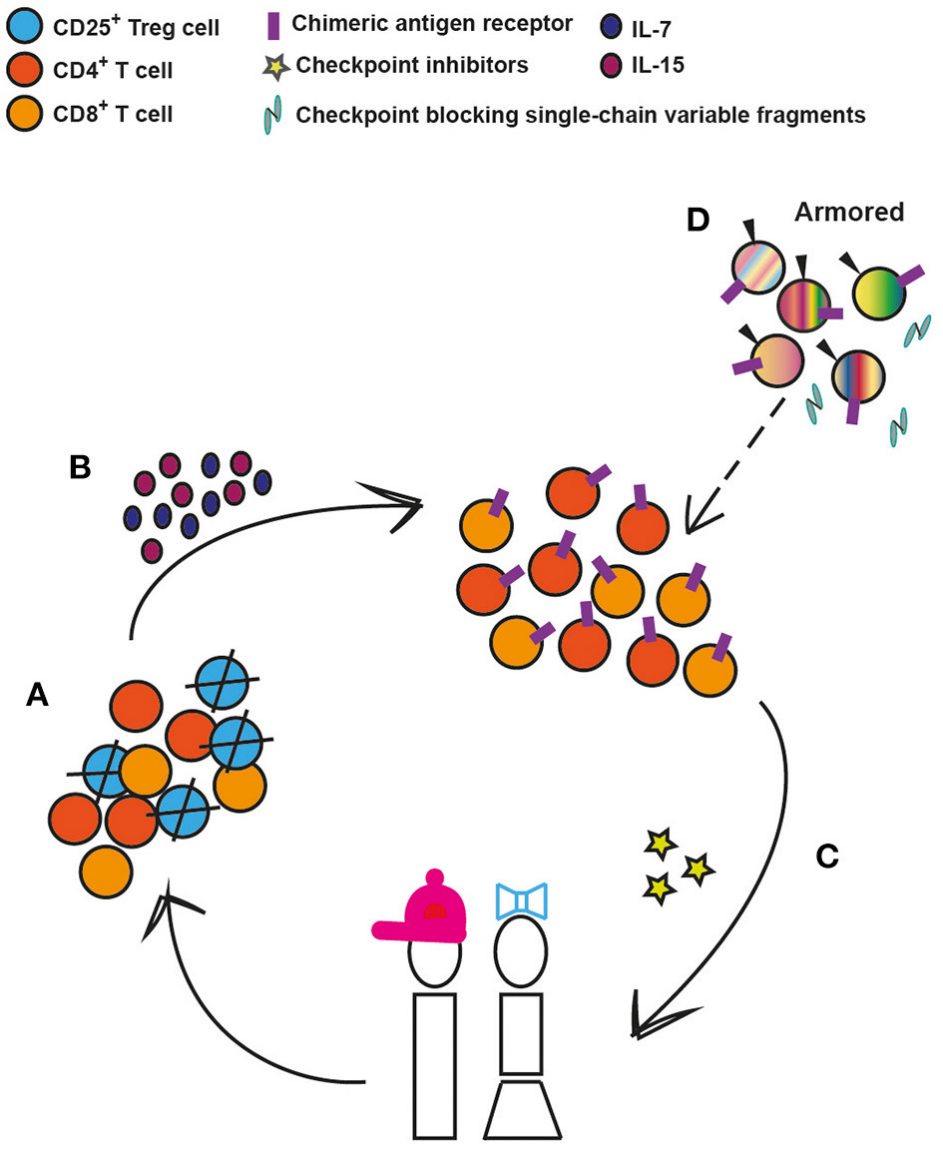
Conceivable strategies to enhance CAR T-cell efficacy and persistence in pediatric ALL. **(A)** Depletion of regulatory T cells (Tregs) prior to chimeric antigen receptor engineering. **(B)** Provision of proliferation and survival factors for T cells. **(C)** CAR T cells can be co-administered with immunomodulatory agents such as check-point inhibitors that target PD-1 or CTLA-4 pathways. **(D)** Armored CAR T cells are genetically modified to, along with the CAR, secrete cytokines, such as Il-7, IL-12, and IL-15, or to express immunomodulatory ligands such as CD40L or to secrete PD-1 blocking antibodies. These 4th generation cells can in the future replace the earlier-generation CAR T cells (depicted by dashed arrow).

Today, it is not yet clear why certain pediatric patients respond to specific types of immunotherapies, while others do not. As we have highlighted, there are major differences in T cell populations in blood and tissue, particularly the markedly higher Treg to effector T cell ratios across the tissues of younger children compared to adults (12) that may influence response to therapy. These findings have been corroborated with recent murine studies on solid tumors showing that Tregs utilize IL-10 and IL-35 to exhaust CD8^+^ T cells (90, 91). In keeping with that CXCL8 and ROS impact cancer progression and T cell survival, respectively (92, 93), and that T cell expansion capability is a predictor of successful clinical expansion (94), selection of T cell subsets ahead of CAR T cell engineering could be a strategy to move the field forward (Figure 2). We envisage that the CD25^+^ T cell subset, containing the CD4^+^ effector Tregs, could be depleted prior to CAR T cell engineering without any significant risk for harmful autoimmunity as previously shown in humans (95, 96) and that the addition of IL-7 and IL-15 thereafter could optimize the *ex vivo* expansion of pediatric CAR T cells (69). Furthermore, there are interesting ongoing studies combining immune check-point inhibitors targeting PD-1 or CTLA-4 pathways, with CD19-CAR T cells or bi-specific T cells engagers [reviewed in (97)], which should also be considered in pediatric clinical trials. Lastly, treatment efficacy can be further enhanced through additional genetic modifications of next-generation, CAR T cells. These armored CAR T cells are genetically equipped to express cytokines, such as IL-7, IL-12, and IL-15, surface ligands, such as CD40L, or to secrete single-chain variable antibody fragments that block the PD-1/PD-1L pathway (97–99), which improve cell persistence and modulate the activity of other endogenous cells to favor T-cell mediated killing rather than suppression. All these approaches are likely to be applicable for optimization of CAR T cell efficacy and persistence in the pediatric ALL setting (Figure 2).

In light of above it is necessary for future trials, to plan for follow-up of children who have received immunotherapy and become long term survivors. Firstly, it is unknown how the naïve and memory T cell pool is affected by conventional chemotherapy at various tissue sites over time. There are findings from non-human primate studies that show differential damage to the memory B-cell compartment by anti-cancer drugs according to the type of secondary lymphoid tissue (100) but equivalent data on the adaptive immune cells of humans remain to be elucidated. Lastly, there is, as yet little data on T cell recall responses in childhood cancer survivors. From our experiences in the clinic, we know that these children suffer from re-activation of herpesviruses and invasive fungal infections during ongoing therapy. Longitudinal data in this regard (especially following T cell-based immunotherapies) to assess the consequence of intense T cell activation on immunosenescence, is warranted in survivors of childhood cancer.

## Author Contributions

AN and SS-H: conceptualization, literature evaluation, figure preparation, funding acquisition, and writing up the paper. ES-E: literature evaluation, funding acquisition, and original draft writing. All authors made a substantial intellectual contribution and approved the manuscript for publication.

## Conflict of Interest

The authors declare that the research was conducted in the absence of any commercial or financial relationships that could be construed as a potential conflict of interest.
